# S13. DO PATIENTS WITH RECENT-ONSET DEPRESSION DIFFER CLINICALLY AND NEUROBIOLOGICALLY FROM DEPRESSED PATIENTS WITH A CLINICAL HIGH-RISK STATE FOR PSYCHOSIS?

**DOI:** 10.1093/schbul/sby018.800

**Published:** 2018-04-01

**Authors:** Rachel Upthegrove, Renate Reniers, Pavan Mallikarjun, Eva Meisenzahl, Katharine Chisholm, Stefan Borgwardt, Stephan Ruhrmann, Raimo Salokangas, Paolo Brambilla, Stephen Wood, Nikolaos Koutsouleris, Paris Alexandros Lalousis

**Affiliations:** 1 University of Birmingham; 2 Ludwig Maximilians University; 3 University of Basel; 4 University Hospital, University of Cologne; 5 University of Turku; 6 University of Milan; 7 Orygen, the National Centre of Excellence in Youth Mental Health

## Abstract

**Background:**

Major depressive disorder (MDD) is one of the most common mental disorders, with a lifetime prevalence of 14.6%. The impact of depression is considerable; poor social and economic functioning and significant life limitations [1]. Depression is also the most common co-morbidity seen with other mental disorders. The prevalence of depressive disorder in schizophrenia has been reported to be around 40% [2]. When examining very early phases of illness, in groups identified as at clinical high risk (CHR) for psychosis, high rates of ‘co-morbid’ axis one diagnoses are reported, with over 50% reaching criteria for a depressive disorder. Those people with schizophrenia send depression are significantly more likely to relapse, to be a safety concern (be arrested, victimized or suicidal), have greater substance-related problems and poorer recovery [2]. In addition, depression has been linked to increased risk of transition from CHR to FEP, suggesting that in this group depression also indicates a poorer outcome [3]. Currently, the diagnosis of depression is based on the phenomenological evaluation of symptoms and behavior. However, there remains significant debate around the heterogeneity of depressive symptoms and their function as prognostic indicators [4]. Neuroimaging holds “diagnostic potential” for depression [5]. However, studies show that brain alterations are often small and reliability is difficult, and there has been no neuroimaging investigation of depression as a co-morbid diagnosis. We aim to further understand the symptom profile of depression in emerging mental disorders, including in the clinical high risk group (CHR) and recent onset psychosis (ROP) as compared to those with recent onset depression (ROD). This has important implications for the accurate identification of a potentially malleable target for treatment, and indeed development of novel therapeutic options. We also aim to explore the ability of brain imagining (structural MRI) to add accuracy to the classification prediction models

**Methods:**

Data from the PRONIA study, an EUFP7 funded 8 center study recruiting ROD, CHR and ROP participants will be presented. Analysis will include demographic information and BDI-II (Beck Depression Inventory), CAARMS (Comprehensive Assessment of the At Risk Mental State), SANS (Scale for the assessment of negative symptoms) total score PANSS (Positive and negative Symptom Score) and SPI-A together with structural MRI imaging. We will report descriptive detail from the PRONIA discovery sample (n716), machine learning classification with Neurominer® and VBM analysis of sMRI scans across groups.

**Results:**

Data from BDI-II symptom endorsement suggests a ‘classical depression phenotype’ corresponding to Becks ‘cognitive triad’; “life is pointless, future hopeless, self as worthless” may separate depression in ROD from ROP, with other symptoms potentially able to separate ROP from ROD. In classification, a 65% sensitivity and specificity are found. Data will also be presented on the CHR group and their alignment, together with VBM analysis for structural MRI examining correlates with highly weighted classifying symptoms in and across all three groups.

**Discussion:**

When given early in the course of illness, interventions have the greatest potential impact, and characterization and accurate diagnosis of depression in emerging mental disorders is an important goal. This study suggests it may be possible to accurately identify depression in different diagnostic categories, including major depressive disorder, psychosis and clinical high risk, and that neuroimaging holds potential to add to diagnostic accuracy in complex co-morbid disorders.

